# Supplementary material for: Antifragility Predicts the Robustness and Evolvability of Biological Networks through Multi-Class Classification with a Convolutional Neural Network
Source: Entropy (Basel). 2020 Sep 4;22(9):986. doi: 10.3390/e22090986 (PMC7597304; doi:10.3390/e22090986)
Supplement: Supplementary file 1 [file entropy-22-00986-s001.pdf]

Supplementary Material

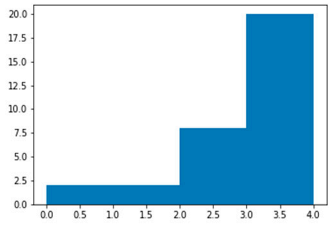

cortical

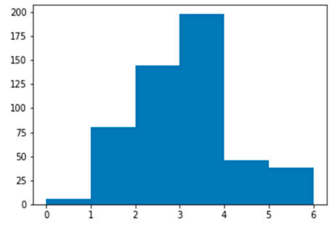

cycle-cdk

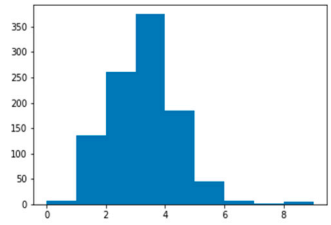

core-cell-cycle

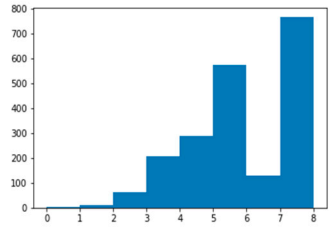

toll-drosophila

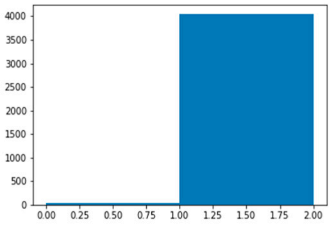

metabolic

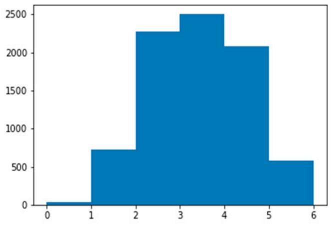

l-arabinose-operon

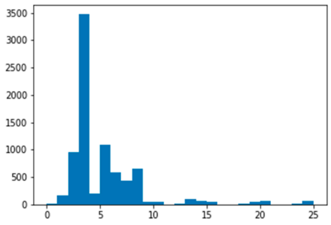

lac-operon-bistability

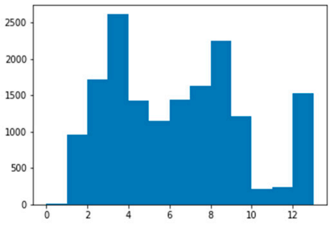

arabidopsis

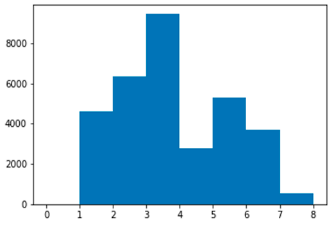

anemia

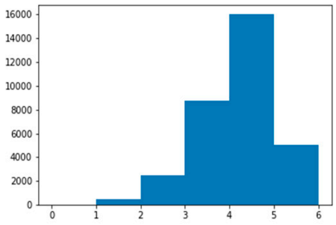

cardiac

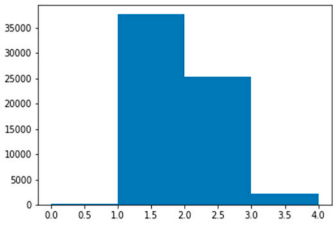

bt474-ErbB

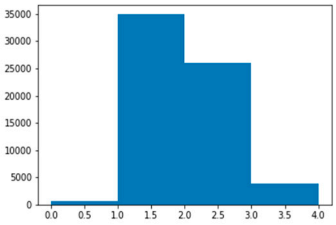

skbr3-short

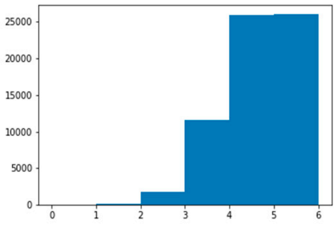

neurotransmitter

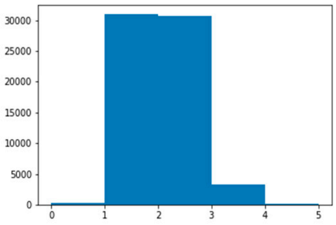

hcc1954-ErbB

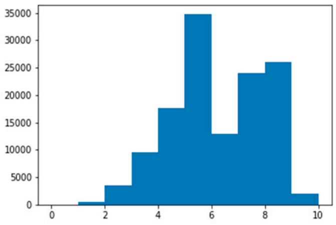

body-drosophila

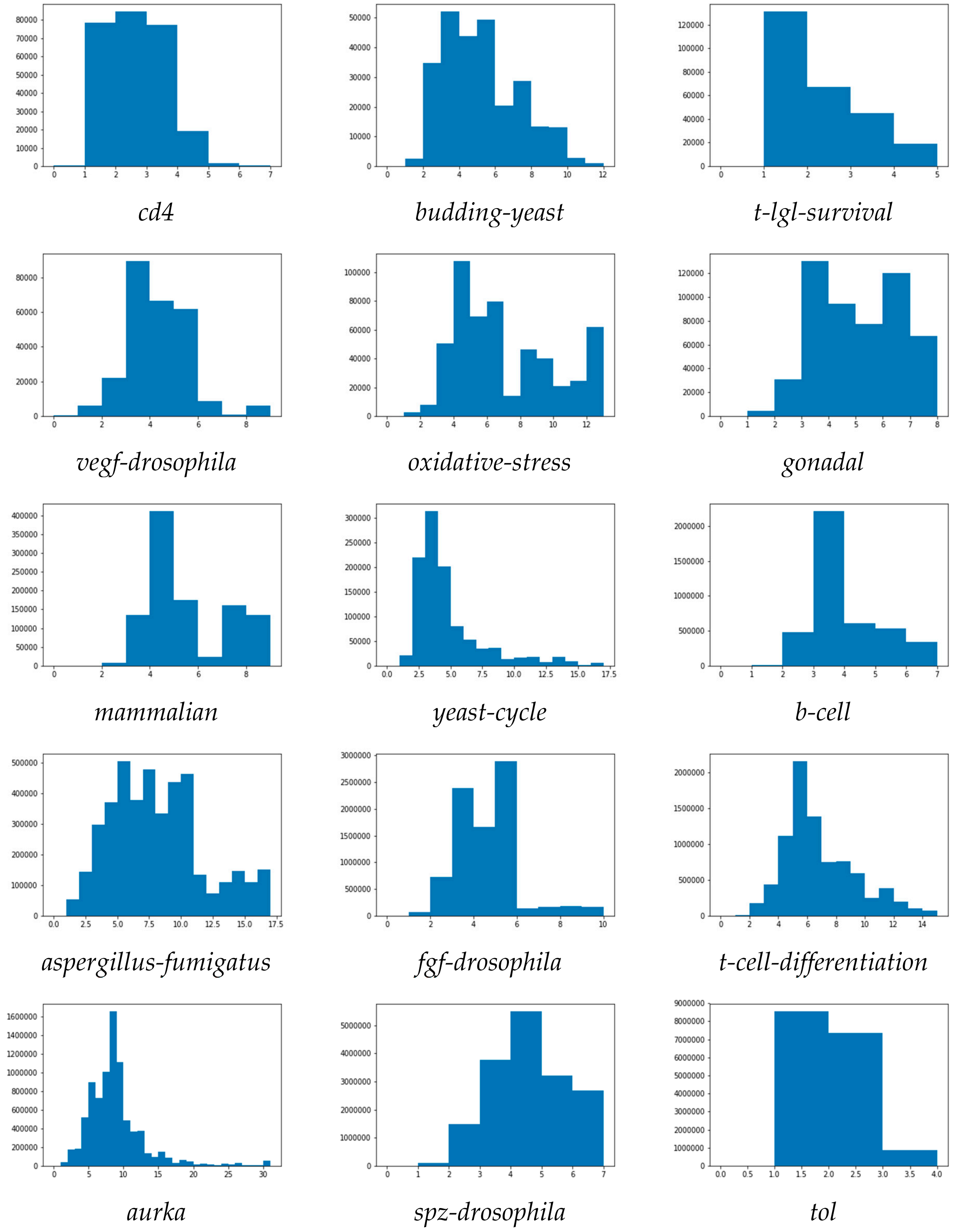
cd4
budding-yeast
t-lgl-survival
vegf-drosophila
oxidative-stress
gonadal
mammalian
yeast-cycle
b-cell
aspergillus-fumigatus
fgf-drosophila
t-cell-differentiation
aurka
spz-drosophila
tol

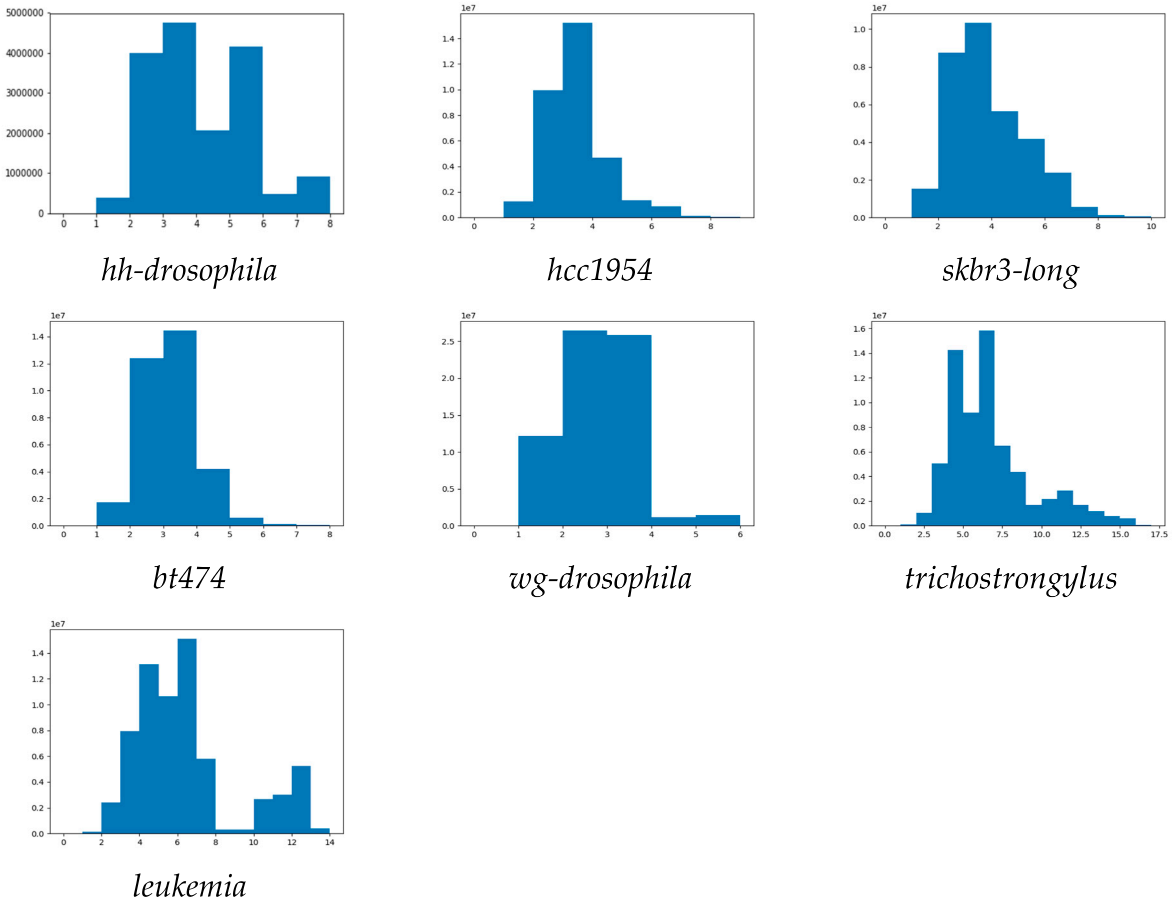

Figure S1. Histograms of transient lengths: how many state transitions all state configurations go through until they reach attractors. For the 37 biological networks, the maximum is 31 in *aurka*, which means that every state configuration of all biological networks converges to attractors within 32 state transitions. This implies that the basins of attraction of biological networks tend to be relatively shallow.

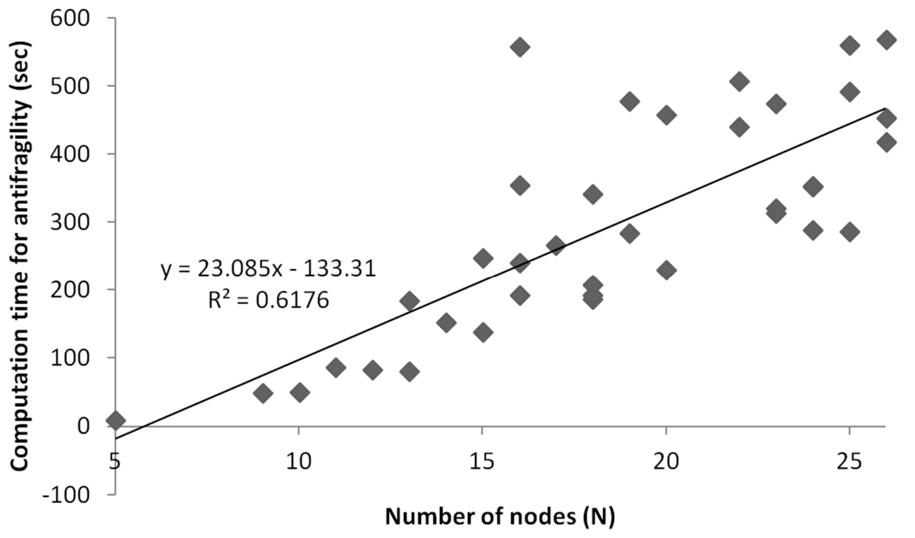

Figure S2. Time complexity of calculating antifragility. It linearly increases with the number of nodes (N).

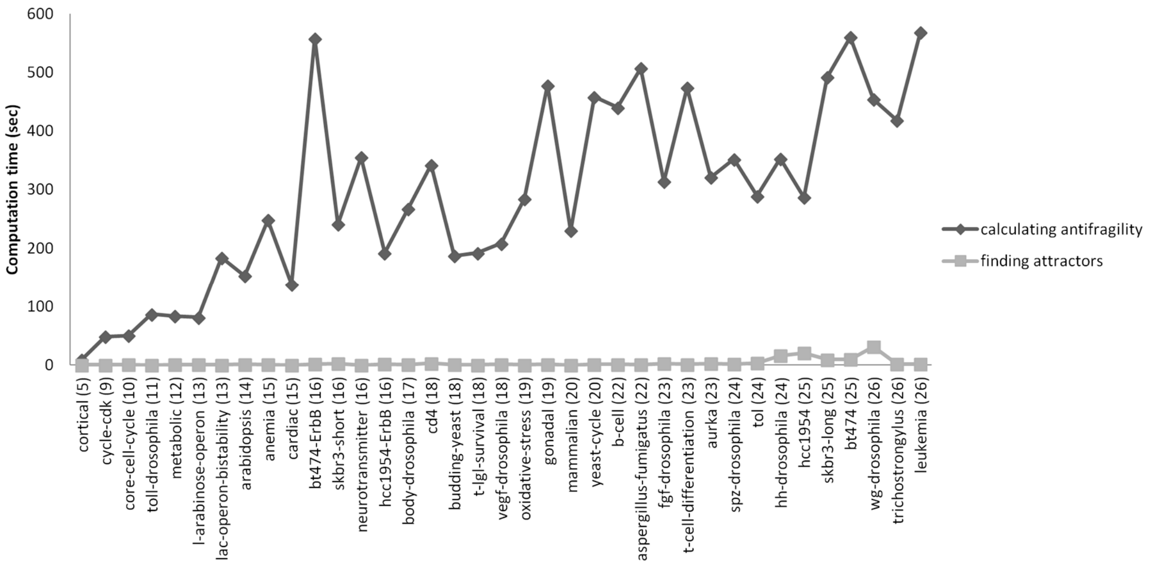

Figure S3. Comparison of computation time between calculating antifragility and finding attractors for the 37 biological networks.
